# 1,2,4-Trimeth­oxy­dibenzo[*b*,*d*]furan-3-ol

**DOI:** 10.1107/S1600536810044417

**Published:** 2010-11-06

**Authors:** S. Yousuf, A. Latif, M. Arfan, M. I. Choudhary

**Affiliations:** a H.E.J. Research Institute of Chemistry, International Center for Chemical and Biological Sciences, University of Karachi, Karachi 75270, Pakistan; b Institute of Chemical Sciences, University of Peshawar, Peshawar, K.P.K., Pakistan

## Abstract

The title compound, C_15_H_14_O_5_, is a natural product, isolated from *Sorbus lanata* Syn. *Pyrus lanata* (D. Don) found in Pakistan. The compound is composed of three spiro-fused rings. The dihedral angle between the mean planes of the benzene rings is 4.81 (13)°. The meth­oxy groups are oriented at dihedral angles of 74.44 (14), 83.0 (2) and 66.3 (2)° with respect to the planes of the benzene rings to which they are attached. The mol­ecule is consolidated by three intra­molecular O—H⋯O and C—H⋯O hydrogen bonds. In the crystal, mol­ecules are linked by inter­molecular O—H⋯O hydrogen bonds, forming infinite chains along the *b* axis.

## Related literature

The title compound was previously reported from a perry pear tree *Pyrus communis*, see: Kemp *et al.* (1983 ▶). For the structure of dibenzofuran, see: Dideberg *et al.* (1972 ▶).
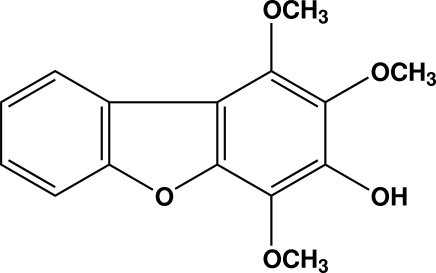


## Experimental

### 

#### Crystal data

C_15_H_14_O_5_
*M* *_r_* = 274.26
Monoclinic, 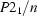

*a* = 10.422 (3) Å
*b* = 9.075 (3) Å
*c* = 15.007 (4) Å
β = 106.378 (7)°
*V* = 1361.8 (6) Å^3^
*Z* = 4
Mo *K*α radiation
μ = 0.10 mm^−1^
*T* = 298 K
0.28 × 0.13 × 0.09 mm

#### Data collection

Bruker SMART APEX CCD area-detector diffractometer
Absorption correction: multi-scan (*SADABS*; Bruker, 2000 ▶) *T*_min_ = 0.972, *T*_max_ = 0.991
7822 measured reflections
2534 independent reflections
1659 reflections with *I* > 2σ(*I*)
*R*_int_ = 0.038

#### Refinement

*R*[*F* ^2^ > 2σ(*F* ^2^)] = 0.050
*wR*(*F* ^2^) = 0.134
*S* = 1.04
2534 reflections
181 parameters
H-atom parameters constrained
Δρ_max_ = 0.25 e Å^−3^
Δρ_min_ = −0.23 e Å^−3^

### 

Data collection: *SMART* (Bruker, 2000 ▶); cell refinement: *SAINT* (Bruker, 2000 ▶); data reduction: *SAINT*; program(s) used to solve structure: *SHELXS97* (Sheldrick, 2008 ▶); program(s) used to refine structure: *SHELXL97* (Sheldrick, 2008 ▶); molecular graphics: *SHELXTL* (Sheldrick, 2008 ▶); software used to prepare material for publication: *SHELXTL*, *PARST* (Nardelli, 1995 ▶) and *PLATON* (Spek, 2009 ▶).

## Supplementary Material

Crystal structure: contains datablocks global, I. DOI: 10.1107/S1600536810044417/pv2347sup1.cif

Structure factors: contains datablocks I. DOI: 10.1107/S1600536810044417/pv2347Isup2.hkl

Additional supplementary materials:  crystallographic information; 3D view; checkCIF report

## Figures and Tables

**Table 1:** Hydrogen-bond geometry (Å, °)

*D*—H⋯*A*	*D*—H	H⋯*A*	*D*⋯*A*	*D*—H⋯*A*
O3—H3*A*⋯O4	0.82	2.32	2.738 (2)	113
C13—H13*B*⋯O4	0.96	2.49	3.092 (4)	121
C15—H15*B*⋯O3	0.96	2.56	3.108 (3)	117
O3—H3*A*⋯O2^i^	0.82	1.97	2.742 (2)	156

Symmetry code: (i) 
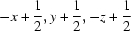
.

**Fig. 1.**
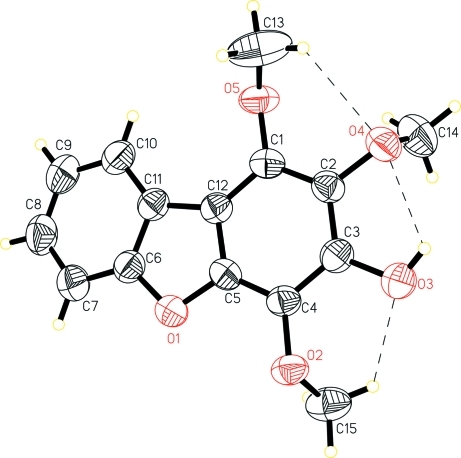
The molecular structure of the title molecule with displacement ellipsoids drawn at 50% probability level. The dashed lines indicate intramolecular hydrogen bonds.

**Fig. 2.**
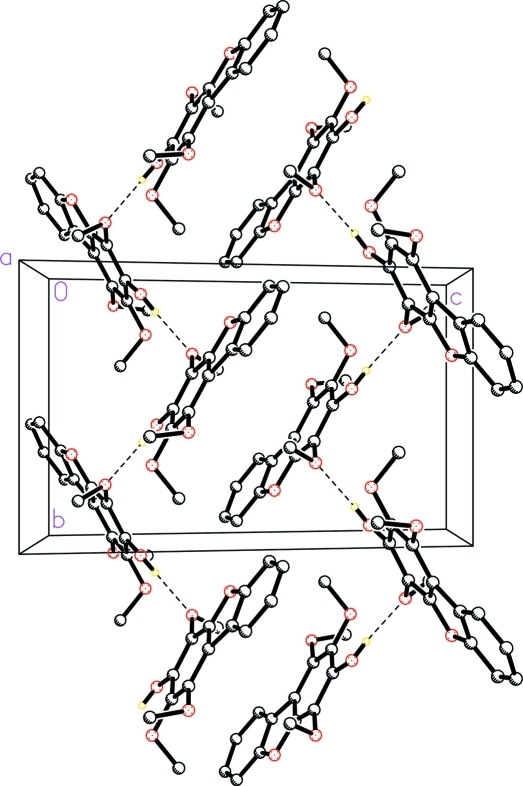
A packing diagram of the title compound showing hydrogen bonds as dashed lines; the hydrogen atoms not involved in bonding have been excluded for clarity.

## supplementary crystallographic information

### Comment

*Sorbus lanata* (D. Don) Schauer is an important medicinal plant, commonly found in Pakistan, Nepal and India. The title compound (Fig. 1) is a dibenzofuran derivative which has been previously reported from perry pear tree *Pyrus communis* (Kemp *et al.*, 1983). It is composed of three spiro fused rings A (C1–C5/C12), B (O1/C5/C6/C11/C12), and C (C6–C11). The dihedral angles between the mean-planes of the rings A/B, B/C and A/C are 2.62 (14), 2.27 (16) and 4.81 (13) °, respectively, reflecting significant deviation from planarity of the fused ring system. The methoxy groups O2/C15, O4/C14 and O/C13 are oriented with respect to the plane of the phenyl ring (C1–C5/C12) at angles 74.44 (14), 82.95 (19) and 66.3 (2)°, respectively. The molecular structure is stabilized by three intramolecular hydrogen bonds O3—H3A···O4, C13—H13B···O4 and C15—H15B···O3. In the crystal structure, the molecules are linked by the O3—H3A···O2 intermolecular hydrogen bonds to form infinite zigzag chains parallel to the *b*-axis (Fig. 2 and Table 1).

The crystal structure of dibenzofuran has been reported (Dideberg *et al.*, (1972).

### Experimental

The dried and crushed wood of *Sorbus lanata *(12 kg) was subjected to cold extraction with methanol. The methanolic extract (750 g) was suspended in water and successively partitioned with hexane, ethylacetate and butanol. The ethylacetate fraction (180 g) was further defatted with hexane several times. The defatted ethylacetate fraction (150 g) was subjected to column chromatography on silica gel [Merck Silica gel60 (0.063-0.200 mm), 9 x 50 cm]. The column was first eluted with hexane-ethylacetate (100:0 → 0:100) and then with dichloromethane-methanol (98:2 → 90:10) as solvent systems. A total of 11 fractions, LS-4 (1 g), LS-5 (2.7 g), LS-11 (15 g), LS-18 (27 g), LS-25 (45 g), LS-46 (20 g),LS-60 (37 g), LS-62 (40 g), LS-73 (35 g), LS-81 (10 g) and LS-89 (8 g) were obtained according to their TLC profiles. Fraction LS-11 contained a white solid insoluble in methanol. The solid material was filtered and the filtrate was concentrated and further subjected to column chromatography on silica gel [Merck Silica gel 60 (0.063-0.200 mm), 4 x 30 cm] using hexane-dichloromethane (100:0 → 0:100) as solvent system and as a result 10 fractions LS-1102, LS-1105, LS-1111, LS-1126, LS-1158, LS-1167, LS-1177, LS-1187, LS-1189 and LS-1199 were obtained. Fraction LS-1126 contained colourless crystals of various sizes and were separated from the solution by decantation. The crystals were washed with hexane several times. To obtain pure and larger crystals, these crystals were re-grown in a mixture of hexane-acetone (4:1) and afford colorles needles (80 mg).

### Refinement

H atoms on the C of methyl, methine and oxygen were positioned geometrically with C—H = 0.96, 0.93 and 0.82 Å, respectively, and constrained to ride on their parent atoms with *U*_iso_(H) = 1.2*U*_eq_(CH) and 1.5*U*_eq_(CH_3_ and OH).

### Crystal data

**Table:**

C_15_H_14_O_5_	*F*(000) = 576
*M**_r_* = 274.26	*D*_x_ = 1.338 Mg m^−^^3^
Monoclinic, *P*2_1_/*n*	Mo *K*α radiation, λ = 0.71073 Å
Hall symbol: -P 2yn	Cell parameters from 1214 reflections
*a* = 10.422 (3) Å	θ = 2.1–25.5°
*b* = 9.075 (3) Å	µ = 0.10 mm^−^^1^
*c* = 15.007 (4) Å	*T* = 298 K
β = 106.378 (7)°	Plate, colorless
*V* = 1361.8 (6) Å^3^	0.28 × 0.13 × 0.09 mm
*Z* = 4	

### Data collection

**Table:**

Bruker SMART APEX CCD area-detector diffractometer	2534 independent reflections
Radiation source: fine-focus sealed tube	1659 reflections with *I* > 2σ(*I*)
graphite	*R*_int_ = 0.038
ω scans	θ_max_ = 25.5°, θ_min_ = 2.1°
Absorption correction: multi-scan (*SADABS*; Bruker, 2000)	*h* = −12→8
*T*_min_ = 0.972, *T*_max_ = 0.991	*k* = −10→10
7822 measured reflections	*l* = −15→18

### Refinement

**Table:**

Refinement on *F*^2^	Primary atom site location: structure-invariant direct methods
Least-squares matrix: full	Secondary atom site location: difference Fourier map
*R*[*F*^2^ > 2σ(*F*^2^)] = 0.050	Hydrogen site location: inferred from neighbouring sites
*wR*(*F*^2^) = 0.134	H-atom parameters constrained
*S* = 1.04	*w* = 1/[σ^2^(*F*_o_^2^) + (0.0588*P*)^2^ + 0.185*P*] where *P* = (*F*_o_^2^ + 2*F*_c_^2^)/3
2534 reflections	(Δ/σ)_max_ < 0.001
181 parameters	Δρ_max_ = 0.25 e Å^−^^3^
0 restraints	Δρ_min_ = −0.23 e Å^−^^3^

### Special details

**Table:**

Geometry. All e.s.d.'s (except the e.s.d. in the dihedral angle between two l.s. planes) are estimated using the full covariance matrix. The cell e.s.d.'s are taken into account individually in the estimation of e.s.d.'s in distances, angles and torsion angles; correlations between e.s.d.'s in cell parameters are only used when they are defined by crystal symmetry. An approximate (isotropic) treatment of cell e.s.d.'s is used for estimating e.s.d.'s involving l.s. planes.
Refinement. Refinement of *F*^2^ against ALL reflections. The weighted *R*-factor *wR* and goodness of fit *S* are based on *F*^2^, conventional *R*-factors *R* are based on *F*, with *F* set to zero for negative *F*^2^. The threshold expression of *F*^2^ > σ(*F*^2^) is used only for calculating *R*-factors(gt) *etc*. and is not relevant to the choice of reflections for refinement. *R*-factors based on *F*^2^ are statistically about twice as large as those based on *F*, and *R*- factors based on ALL data will be even larger.

### Fractional atomic coordinates and isotropic or equivalent isotropic displacement parameters (Å^2^)

**Table:**

	*x*	*y*	*z*	*U*_iso_*/*U*_eq_	
O1	−0.04296 (15)	0.68527 (16)	0.05209 (11)	0.0506 (4)	
O2	0.21877 (14)	0.79638 (16)	0.14577 (10)	0.0486 (4)	
O3	0.22888 (14)	1.06736 (17)	0.23002 (11)	0.0583 (5)	
H3A	0.2202	1.1346	0.2644	0.087*	
O4	0.00148 (15)	1.21507 (17)	0.23400 (11)	0.0547 (5)	
O5	−0.25302 (15)	1.08945 (18)	0.14506 (11)	0.0574 (5)	
C10	−0.3847 (2)	0.7966 (3)	0.01571 (18)	0.0567 (7)	
H1A	−0.4312	0.8738	0.0331	0.068*	
C9	−0.4521 (3)	0.6846 (3)	−0.04119 (19)	0.0657 (8)	
H2A	−0.5450	0.6865	−0.0618	0.079*	
C8	−0.3837 (3)	0.5697 (3)	−0.06806 (19)	0.0673 (8)	
H3B	−0.4318	0.4967	−0.1069	0.081*	
C7	−0.2460 (3)	0.5609 (3)	−0.03862 (18)	0.0599 (7)	
H4A	−0.1997	0.4836	−0.0560	0.072*	
C6	−0.1813 (2)	0.6732 (2)	0.01794 (16)	0.0482 (6)	
C5	−0.0212 (2)	0.8160 (2)	0.10136 (15)	0.0439 (6)	
C4	0.1037 (2)	0.8724 (2)	0.14437 (15)	0.0431 (5)	
C3	0.1077 (2)	1.0079 (2)	0.18832 (15)	0.0455 (6)	
C2	−0.0113 (2)	1.0811 (2)	0.18854 (15)	0.0442 (5)	
C1	−0.1354 (2)	1.0187 (2)	0.14702 (15)	0.0449 (6)	
C12	−0.1400 (2)	0.8844 (2)	0.10160 (15)	0.0427 (5)	
C11	−0.2457 (2)	0.7908 (2)	0.04626 (16)	0.0463 (6)	
C13	−0.2808 (3)	1.1053 (5)	0.2305 (2)	0.1109 (13)	
H13A	−0.3646	1.1555	0.2214	0.166*	
H13B	−0.2110	1.1614	0.2720	0.166*	
H13C	−0.2860	1.0097	0.2567	0.166*	
C14	−0.0108 (3)	1.3386 (3)	0.1722 (2)	0.0771 (9)	
H14A	−0.0017	1.4285	0.2071	0.116*	
H14B	−0.0969	1.3363	0.1270	0.116*	
H14C	0.0579	1.3336	0.1411	0.116*	
C15	0.2906 (3)	0.8538 (3)	0.08552 (18)	0.0655 (8)	
H15A	0.3688	0.7949	0.0905	0.098*	
H15B	0.3168	0.9536	0.1028	0.098*	
H15C	0.2345	0.8517	0.0226	0.098*	

### Atomic displacement parameters (Å^2^)

**Table:**

	*U*^11^	*U*^22^	*U*^33^	*U*^12^	*U*^13^	*U*^23^
O1	0.0472 (10)	0.0450 (9)	0.0594 (10)	0.0019 (7)	0.0146 (8)	−0.0062 (7)
O2	0.0421 (9)	0.0491 (9)	0.0562 (10)	0.0098 (7)	0.0166 (7)	0.0073 (7)
O3	0.0439 (10)	0.0597 (10)	0.0667 (12)	−0.0015 (8)	0.0083 (8)	−0.0159 (8)
O4	0.0566 (11)	0.0511 (10)	0.0555 (10)	0.0012 (8)	0.0147 (8)	−0.0124 (8)
O5	0.0458 (10)	0.0680 (11)	0.0609 (11)	0.0133 (8)	0.0194 (8)	−0.0070 (8)
C10	0.0462 (15)	0.0612 (16)	0.0641 (17)	−0.0034 (12)	0.0181 (12)	0.0032 (13)
C9	0.0508 (16)	0.0769 (19)	0.0667 (18)	−0.0146 (14)	0.0122 (13)	−0.0021 (15)
C8	0.0666 (19)	0.0657 (17)	0.0675 (18)	−0.0206 (14)	0.0153 (14)	−0.0127 (14)
C7	0.0606 (18)	0.0550 (15)	0.0641 (17)	−0.0074 (12)	0.0175 (13)	−0.0097 (13)
C6	0.0434 (14)	0.0497 (13)	0.0511 (14)	−0.0022 (10)	0.0128 (11)	0.0003 (11)
C5	0.0490 (14)	0.0375 (12)	0.0450 (13)	0.0036 (10)	0.0131 (10)	0.0008 (10)
C4	0.0400 (13)	0.0441 (12)	0.0444 (13)	0.0065 (10)	0.0107 (10)	0.0045 (10)
C3	0.0417 (14)	0.0490 (13)	0.0436 (13)	0.0002 (10)	0.0083 (10)	0.0009 (10)
C2	0.0464 (14)	0.0445 (12)	0.0406 (13)	0.0019 (10)	0.0105 (10)	−0.0040 (10)
C1	0.0453 (14)	0.0469 (13)	0.0436 (13)	0.0077 (10)	0.0146 (10)	0.0028 (10)
C12	0.0412 (13)	0.0440 (12)	0.0424 (13)	0.0020 (10)	0.0112 (10)	0.0029 (10)
C11	0.0454 (14)	0.0486 (13)	0.0460 (13)	−0.0016 (10)	0.0148 (10)	0.0041 (11)
C13	0.097 (3)	0.164 (4)	0.093 (3)	0.034 (2)	0.062 (2)	0.012 (2)
C14	0.096 (2)	0.0492 (16)	0.086 (2)	0.0019 (14)	0.0267 (17)	−0.0016 (15)
C15	0.0640 (18)	0.0750 (18)	0.0657 (18)	0.0167 (14)	0.0316 (14)	0.0152 (15)

### Geometric parameters (Å, °)

**Table:**

O1—C5	1.383 (2)	C7—H4A	0.9300
O1—C6	1.391 (3)	C6—C11	1.390 (3)
O2—C4	1.379 (3)	C5—C4	1.378 (3)
O2—C15	1.425 (3)	C5—C12	1.386 (3)
O3—C3	1.353 (2)	C4—C3	1.390 (3)
O3—H3A	0.8200	C3—C2	1.408 (3)
O4—C2	1.382 (2)	C2—C1	1.389 (3)
O4—C14	1.438 (3)	C1—C12	1.391 (3)
O5—C1	1.377 (3)	C12—C11	1.451 (3)
O5—C13	1.399 (3)	C13—H13A	0.9600
C10—C9	1.385 (3)	C13—H13B	0.9600
C10—C11	1.391 (3)	C13—H13C	0.9600
C10—H1A	0.9300	C14—H14A	0.9600
C9—C8	1.385 (4)	C14—H14B	0.9600
C9—H2A	0.9300	C14—H14C	0.9600
C8—C7	1.380 (4)	C15—H15A	0.9600
C8—H3B	0.9300	C15—H15B	0.9600
C7—C6	1.375 (3)	C15—H15C	0.9600
			
C5—O1—C6	105.14 (16)	O4—C2—C3	117.00 (19)
C4—O2—C15	114.67 (17)	C1—C2—C3	121.1 (2)
C3—O3—H3A	109.5	O5—C1—C2	122.0 (2)
C2—O4—C14	112.89 (18)	O5—C1—C12	119.4 (2)
C1—O5—C13	116.2 (2)	C2—C1—C12	118.5 (2)
C9—C10—C11	118.4 (2)	C5—C12—C1	119.1 (2)
C9—C10—H1A	120.8	C5—C12—C11	105.73 (19)
C11—C10—H1A	120.8	C1—C12—C11	135.1 (2)
C10—C9—C8	121.3 (2)	C6—C11—C10	118.4 (2)
C10—C9—H2A	119.4	C6—C11—C12	105.7 (2)
C8—C9—H2A	119.4	C10—C11—C12	135.9 (2)
C7—C8—C9	121.6 (2)	O5—C13—H13A	109.5
C7—C8—H3B	119.2	O5—C13—H13B	109.5
C9—C8—H3B	119.2	H13A—C13—H13B	109.5
C6—C7—C8	116.1 (2)	O5—C13—H13C	109.5
C6—C7—H4A	121.9	H13A—C13—H13C	109.5
C8—C7—H4A	121.9	H13B—C13—H13C	109.5
C7—C6—C11	124.2 (2)	O4—C14—H14A	109.5
C7—C6—O1	124.3 (2)	O4—C14—H14B	109.5
C11—C6—O1	111.48 (19)	H14A—C14—H14B	109.5
C4—C5—O1	124.04 (19)	O4—C14—H14C	109.5
C4—C5—C12	124.0 (2)	H14A—C14—H14C	109.5
O1—C5—C12	111.94 (19)	H14B—C14—H14C	109.5
C5—C4—O2	121.6 (2)	O2—C15—H15A	109.5
C5—C4—C3	116.7 (2)	O2—C15—H15B	109.5
O2—C4—C3	121.7 (2)	H15A—C15—H15B	109.5
O3—C3—C4	118.1 (2)	O2—C15—H15C	109.5
O3—C3—C2	121.2 (2)	H15A—C15—H15C	109.5
C4—C3—C2	120.7 (2)	H15B—C15—H15C	109.5
O4—C2—C1	121.91 (19)		
			
C11—C10—C9—C8	−0.5 (4)	C13—O5—C1—C2	−68.4 (3)
C10—C9—C8—C7	0.7 (4)	C13—O5—C1—C12	115.4 (3)
C9—C8—C7—C6	−0.6 (4)	O4—C2—C1—O5	3.0 (3)
C8—C7—C6—C11	0.3 (4)	C3—C2—C1—O5	−178.87 (19)
C8—C7—C6—O1	−178.0 (2)	O4—C2—C1—C12	179.22 (19)
C5—O1—C6—C7	177.2 (2)	C3—C2—C1—C12	−2.6 (3)
C5—O1—C6—C11	−1.3 (2)	C4—C5—C12—C1	0.5 (3)
C6—O1—C5—C4	−177.8 (2)	O1—C5—C12—C1	−178.95 (18)
C6—O1—C5—C12	1.6 (2)	C4—C5—C12—C11	178.1 (2)
O1—C5—C4—O2	−3.8 (3)	O1—C5—C12—C11	−1.3 (2)
C12—C5—C4—O2	176.8 (2)	O5—C1—C12—C5	178.0 (2)
O1—C5—C4—C3	177.83 (19)	C2—C1—C12—C5	1.6 (3)
C12—C5—C4—C3	−1.5 (3)	O5—C1—C12—C11	1.2 (4)
C15—O2—C4—C5	106.2 (2)	C2—C1—C12—C11	−175.2 (2)
C15—O2—C4—C3	−75.5 (3)	C7—C6—C11—C10	−0.1 (4)
C5—C4—C3—O3	−179.0 (2)	O1—C6—C11—C10	178.4 (2)
O2—C4—C3—O3	2.6 (3)	C7—C6—C11—C12	−178.0 (2)
C5—C4—C3—C2	0.5 (3)	O1—C6—C11—C12	0.5 (2)
O2—C4—C3—C2	−177.87 (19)	C9—C10—C11—C6	0.2 (3)
C14—O4—C2—C1	−84.2 (3)	C9—C10—C11—C12	177.3 (2)
C14—O4—C2—C3	97.6 (2)	C5—C12—C11—C6	0.5 (2)
O3—C3—C2—O4	−0.7 (3)	C1—C12—C11—C6	177.5 (2)
C4—C3—C2—O4	179.8 (2)	C5—C12—C11—C10	−176.9 (3)
O3—C3—C2—C1	−178.9 (2)	C1—C12—C11—C10	0.2 (5)
C4—C3—C2—C1	1.6 (3)		

### Hydrogen-bond geometry (Å, °)

**Table:**

*D*—H···*A*	*D*—H	H···*A*	*D*···*A*	*D*—H···*A*
O3—H3A···O4	0.82	2.32	2.738 (2)	113
C13—H13B···O4	0.96	2.49	3.092 (4)	121
C15—H15B···O3	0.96	2.56	3.108 (3)	117
O3—H3A···O2^i^	0.82	1.97	2.742 (2)	156

Symmetry codes:  (i) −*x*+1/2, *y*+1/2, −*z*+1/2.
